# Wavelength-tunable entangled photons from silicon-integrated III–V quantum dots

**DOI:** 10.1038/ncomms10387

**Published:** 2016-01-27

**Authors:** Yan Chen, Jiaxiang Zhang, Michael Zopf, Kyubong Jung, Yang Zhang, Robert Keil, Fei Ding, Oliver G. Schmidt

**Affiliations:** 1 Institute for Integrative Nanosciences, IFW Dresden, Helmholtzstraße 20, 01069 Dresden, Germany; 2 Material Systems for Nanoelectronics, Chemnitz University of Technology, Reichenhainer strasse 70, 09107 Chemnitz, Germany

## Abstract

Many of the quantum information applications rely on indistinguishable sources of polarization-entangled photons. Semiconductor quantum dots are among the leading candidates for a deterministic entangled photon source; however, due to their random growth nature, it is impossible to find different quantum dots emitting entangled photons with identical wavelengths. The wavelength tunability has therefore become a fundamental requirement for a number of envisioned applications, for example, nesting different dots via the entanglement swapping and interfacing dots with cavities/atoms. Here we report the generation of wavelength-tunable entangled photons from on-chip integrated InAs/GaAs quantum dots. With a novel anisotropic strain engineering technique based on PMN-PT/silicon micro-electromechanical system, we can recover the quantum dot electronic symmetry at different exciton emission wavelengths. Together with a footprint of several hundred microns, our device facilitates the scalable integration of indistinguishable entangled photon sources on-chip, and therefore removes a major stumbling block to the quantum-dot-based solid-state quantum information platforms.

## Abstract (web summary)

Deterministic sources of entangled photons are important for photonic quantum networks, but many applications are only possible when their wavelengths are tunable. Here, the authors use on-chip strain engineering to demonstrate such a source with silicon-integrated InAs/GaAs quantum dots.

## Graphical abstract

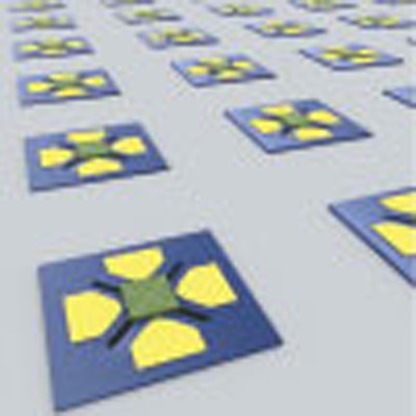

A topical challenge in quantum information processing (QIP) is the generation and manipulation of polarization-entangled photon pairs^1^,^2^. Spontaneous parametric-down-conversion (SPDC) and four-wave-mixing (FWM) have served as the main workhorses for these purposes in the past decade, and the implementation of a fully integrated quantum device is within reach by marrying these sources with chip-scale silicon photonics^3^,^4^,^5^,^6^. However, the generated photons are characterized by Poissonian statistics, that is, one usually does not know when an entangled photon pair is emitted. This fundamentally limits their applications in complex quantum protocols, for example, an event-ready test of Bell's inequality and high-efficiency entanglement purifications, where deterministic operations are much favoured^1^.

The intrinsic limitations of SPDC and FWM processes call for next generation entangled photon sources. III–V semiconductor quantum dots, often referred to as artificial atoms, are among the leading candidates for deterministic quantum light sources. As proposed by Benson *et al*. single quantum dots (QDs) can generate polarization-entangled photon pairs via its biexciton (XX) cascade decay through the intermediate exciton states X, Fig. 1 (ref. 7). In real III–V QDs the anisotropy in strain, composition and shape reduces the QD symmetry to *C*_2v_ or the even lower *C*_1_, leading to the appearance of an energetic splitting between the two bright X states, the so-called fine structure splitting (FSS)^8^. High fidelity to the entangled state 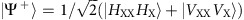, with *H* and *V* denoting the horizontal and vertical polarizations, can be observed only with a vanishing FSS (typically, smaller than the radiative linewidth of ∼1 μeV). The probability of finding such QDs in an as-grown sample is <10^−2^. After extensive efforts by many groups, the elimination of FSS can be achieved by applying rapid thermal annealing^9^, optical Stark effect^10^, magnetic field^11^,^12^, electric field^13^,^14^, and more recently, anisotropic strain fields^15^,^16^ to the QDs. In the past years we have witnessed considerable progress in this field, and entangled photon emissions can be triggered optically^11^,^17^ from single QDs with high brightness (up to 0.12 pair per excitation pulse)^18^ and high indistinguishability (0.86±0.03 for the XX photons)^19^. III–V QDs also possess an important advantage of being compatible with mature semiconductor technology, and electrically triggered entangled photon sources have been successfully demonstrated^16^,^20^.

Armed with these powerful techniques, III–V QDs have the potential to fulfil the ‘wish-list' of a perfect entangled photon source^21^. Among the next goals are the miniaturization and scaling up of the technology. Several important issues need to be considered. First, the FSS of each QD can only be eliminated under particular tuning parameters, and any attempt to manipulate the emission wavelength increases the FSS and spoils the entanglement. This fact undoubtedly restricts the entangled photon emissions at arbitrary wavelengths. The inability to tune the emission wavelength without restoring the FSS, which is unfortunately the common disadvantage associated with all FSS tuning technologies to date, has become a major stumbling block to the QIP applications based on scalable QD sources. Second, as being investigated with SPDC and FWM sources^3^,^4^,^5^, the integration of wavelength-tunable quantum light sources on silicon is arguably one of the most promising choices for on-chip QIP applications^22^.

Here we demonstrate wavelength-tunable entangled photon sources based on III–V QDs integrated on a silicon chip. There are two recent theoretical proposals^23^,^24^ on generating wavelength-tunable entangled photon from QDs, however, the experimental implementations of their proposals are quite challenging. We design and fabricate a device consisting of QD-embedded nanomembranes suspended on a four-legged thin-film PMN-PT ([Pb(Mg_1/3_Nb_2/3_)O_3_]_0.72_[PbTiO_3_]_0.28_) actuator integrated on a silicon substrate. With the combined uniaxial stresses along two orthogonal directions, we are able to keep the FSS strictly below 1 μeV while shifting the exciton wavelength/energy by more than 3,000 times of the QD radiative linewidth. High-fidelity entangled photon emission is demonstrated when the FSS is tuned to below 1 μeV. Therefore wavelength-tunable entangled photons are generated on chip with a single-device footprint of a few hundred microns.

## Results

### Concept of device

**Figure 1:**
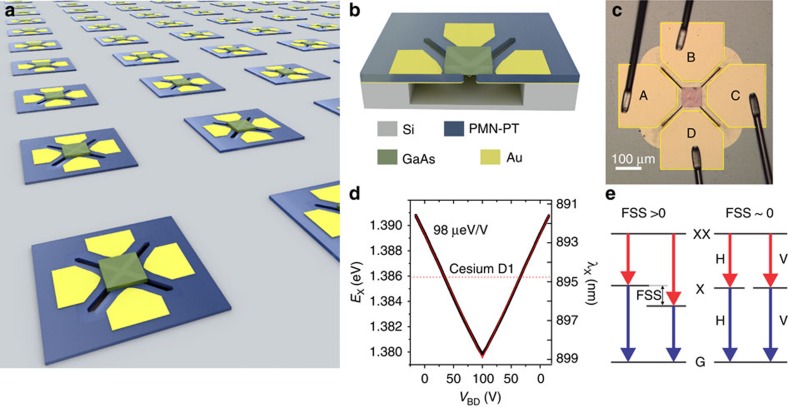
Wavelength-tunable polarization-entangled photon sources integrated on silicon. (**a**) MEMS devices for anisotropic strain engineering of III–V QD-based quantum light sources. Owing to its small footprint and the compatibility with mature semiconductor technologies, large scale on-chip integration is feasible. (**b**) Schematic of the cross section of a single device. Focused ion beam (FIB) cut is used to define trenches on the PMN-PT thin film, and then wet-chemical undercut is used to form four suspended actuation legs. A thin GaAs nanomembrane containing In(Ga)As QDs is transferred onto the suspended region between the four legs. (**c**) Micrograph showing the zoom-in of a completed device. Electrical contacts are made on the four legs A–D. The centre region is a bonded QD-containing nanomembrane. (**d**) Performance of a typical device. The exciton wavelength of a single QD is recorded when the voltage on legs B&D is scanned. The actuation legs contract under positive voltages, leading to the tensile stresses on the nanomembrane and to the red shift of the QD emission. The red solid lines show the linear fit. The dashed line indicates the caesium D1 absorption line. (**e**) Illustration of the FSS in a QD. Polarization-entangled photons are emitted from the XX cascade emission only when the FSS is tuned to near zero.

For the device fabrication we use the industrial transfer printing and die bonding techniques to realize the novel integration of III–V, PMN-PT and Si. Unlike the piezo substrate used in all previous works^15^,^16^,^24^,^25^,^26^,^27^, a 15-μm PMN-PT thin-film bonded on a silicon substrate is employed here to realize novel micro-electromechanical system (MEMS) devices with sophisticated functionalities on chip (Fig. 1, see also Methods section). Arrays of QD-containing GaAs nanomembranes, each 80 × 80 μm^2^ in size, were then transferred onto the PMN-PT MEMS with four actuation legs (Fig. 1a–c). The crystal axes [1–10] and [110] of the GaAs nanomembrane were carefully aligned along the designed stress axes of the actuators. When applying negative (positive) voltages to the electric contacts, the PMN-PT legs expand (contract) in-plane and therefore exert quasi-uniaxial compressive (tensile) stresses to the QDs.

This new device concept has several advantages. First, a controllable anisotropic strain is achieved by the four-legged configuration, which is not possible with any piezo substrate. Second, the use of piezoelectric film alleviates us from high voltages (typically, up to thousands volts) required for the bulk PMN-PT substrate, which is certainly important for on-chip integration. And third, theoretically proposed scalable sources of strain-controlled quantum light sources^23^,^24^ can be realized on chip with such devices.

By sweeping the voltage on only one pair of opposite legs, the exciton emission is shifted over a large range (up to 10 nm in wavelength or 12 meV in energy, Fig. 1d) due to the quasi-uniaxial stresses along the legs. For suitable QDs the exciton emission can be tuned across the caesium D1 line at ∼894.7 nm, which is required for realizing a hybrid quantum memory^28^. To confirm the performance of the device, we have also performed finite-element modelling of our device and two principle stresses (with a magnitude of ∼2 GPa at voltages of 50 V) along orthogonal directions can be identified (not shown here).

**Figure 2:**
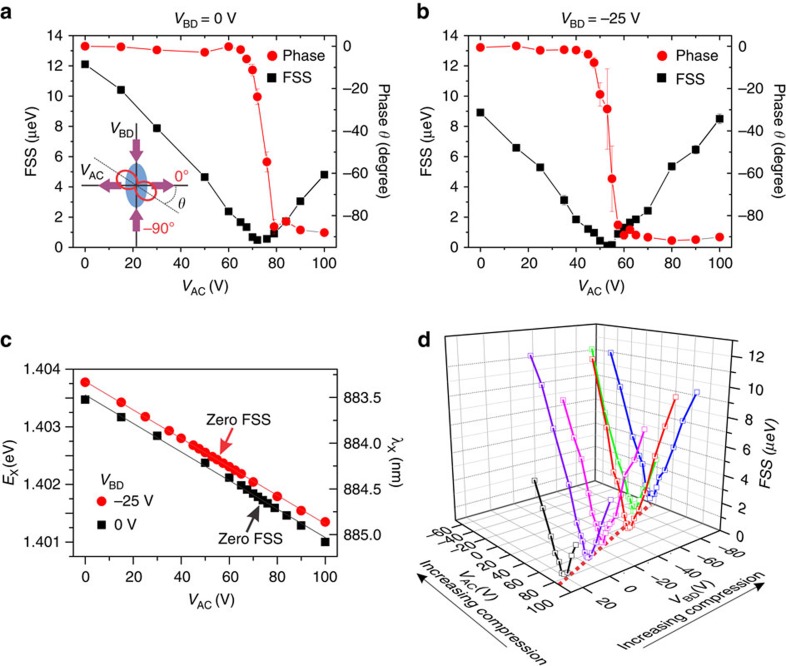
Anisotropic strain engineering of a QD under orthogonal uniaxial stresses. (**a**,**b**) FSS and phase *θ* are plotted as a function of the voltage *V*_AC_ at a fixed voltage *V*_BD_ of 0 and −25 V, respectively. With the increasing voltages on legs A&C, FSS decreases monotonically to around zero and then increases again. And the phase shows an abrupt change from 0 to −90°, when FSS reaches the minimum value. The inset in **a** gives the definition of *θ*. The ellipse indicates an elongated QD with its major axis aligned along a crystallographic direction. The red solid line indicates the exciton polarization. The error bars originate from the fitting process. (**c**) Exciton wavelength is plotted as a function of *V*_AC_ for the two different *V*_BD_. The solid lines are linear fits. The arrows indicate exciton wavelengths at which the FSS are erased. (**d**) The changes in FSS when both *V*_BD_ and *V*_AC_ are scanned. The dashed line on the bottom plane indicates a linear shift of the voltage combination (*V*_AC_*, V*_BD_) at which FSS reaches the minimum values.

Theory^26^,^27^,^29^ predicts that the uniaxial strain tuning behaviour of FSS is determined by the QD principal axis with respect to the uniaxial stress direction. For a QD whose principal axis is closely aligned with the stress direction, the FSS can be effectively eliminated. For typical self-assembled semiconductor QDs the directions of the QD principal axes have a Gaussian-like distribution, and a significant amount^30^ of QDs are closely aligned with the crystallographic directions due to the anisotropic surface adatom diffusion. An example of the FSS tuning behaviour for such an aligned QD is given in Fig. 2a. The uniaxial stress tuning is done by sweeping the voltage *V*_AC_ from 0 to 100 V on one pair of legs, whereas fixing the voltage *V*_BD_ at 0 V on the other pair of legs. With increasing *V*_AC_ the FSS first decreases monotonically to a minimum value and then increases. At *V*_AC_ of ∼73 V the FSS is completely eliminated. The phase *θ*, which indicates the angle (see inset of Fig. 2a) between the exciton polarization and the [1–10] crystallographic direction of GaAs, undergoes a sharp phase change of 90°. This result is in agreement with theoretical predictions^27^.

How does a QD behave when both voltages *V*_AC_ and *V*_BD_ are turned on, that is, under the application of a pair of orthogonal uniaxial stresses? This has never been studied due to the lack of realistic experimental techniques. In Fig. 2b we present the FSS tuning result at a different *V*_BD_ of −25 V. Similar to Fig. 2a we also observe a zero FSS and an abrupt change in *θ* by exactly 90°. The only difference between the two situations (*V*_BD_=0 and −25 V) is the voltage *V*_AC_ at which the FSS is erased. This is an indication of the recovery of QD electronic symmetry at different experimental conditions. It is then interesting to see how the exciton emission wavelength changes, Fig. 2c. With negative (positive) voltages applied, the legs exert uniaxial compressive (tensile) stresses to the QD which causes a blue (red) shift in exciton emission. This is confirmed by sweeping *V*_AC_ from 0 to 100 V, from which we observe a red shift of the emission. The device performs remarkably well and we do not observe any hysteresis in the wavelength tuning, see the linear fit. The effect is similar when changing *V*_BD_ from −25 to 0 V at a fixed *V*_AC_. Therefore we have a high degree of control on the exciton wavelength by using two pairs of actuation legs.

### Wavelength-tunable entangled photons from QDs

Two-dimensional scanning on the two pairs of legs by sweeping both *V*_BD_ and *V*_AC_ is then performed. In Fig. 2d we show the results in a three-dimensional plot. The astonishing result is that, with this four-legged device providing orthogonal uniaxial stresses, multiple zero FSS points with different exciton wavelength *λ*_X_ (energy *E*_X_) can be achieved. At different *V*_BD_, the electronic symmetry of quantum dot can be always recovered by sweeping *V*_AC_ and the FSS is erased. The dashed line on the bottom plane of the plot indicates the combinations of (*V*_AC_ and *V*_BD_) at which the FSS reaches its minimum. A linear relationship is found for the ratio of voltage changes Δ*V*_AC_/Δ*V*_BD_. In terms of the applied stresses (*X, Y*), indeed, an effective two-level model (Supplementary Note 1) for the FSS of QDs with exciton polarization closely aligned to principal stress axes predicts a zero FSS with a linear relationship *ΔX/ΔY* and confirms this experimental finding.

**Figure 3:**
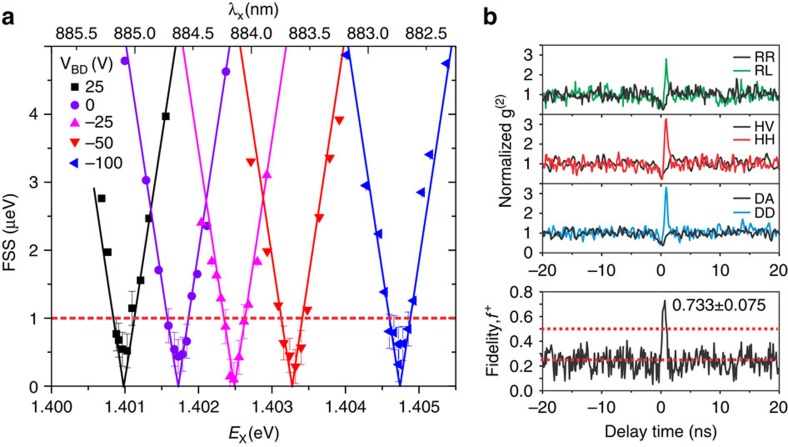
Independent tunability of exciton wavelength and FSS. (**a**) FSS is plotted as a function of the exciton wavelength *λ*_X_ (energy *E*_X_), at different values of *V*_BD_. The solid lines are theoretical fits. In the **k·p** theory we consider the effect of a pair of orthogonal uniaxial stresses applied to an aligned QD. Exciton energy at which FSS ∼0 is tuned by 3.7 meV. The dashed line is a threshold of 1 μeV for the entangled photons generations. For FSS of below 1 μeV, the error bars of ±0.25 μeV are indicated (Methods section). (**b**) Polarization correlation spectroscopy, Methods section, is performed on the biexciton and exciton photons, when the QD FSS is tuned to zero. The normalized coincident counts are given for both co-polarized and cross-polarized photons. We have measured a fidelity *f*^+^ of 0.733±0.075 without any background subtraction. The two dashed lines indicate the threshold of 0.5 for the classically correlated light, and the threshold of 0.25 for the uncorrelated light.

As the independent tunability of exciton wavelength and FSS is a main concern in this work, we plot in Fig. 3a the FSS versus exciton wavelength for different *V*_BD_. For clarity, we show only the FSS range from 0 to 5 μeV. It shows exactly a linear behaviour (represented by the solid line fits) in agreement with a **k.p** analysis, Supplementary Note 1. For self-assembled InAs/GaAs QDs the relationship between the FSS and the attainable entanglement fidelity *f*^*+*^ has been well documented and it is commonly accepted that the entanglement persists for a FSS even up to 3–4 μeV (refs 15, 16, 31). As shown in these reports, tuning the FSS to <1 μeV yields a high fidelity *f*^*+*^ of >0.7. With this novel device, it is clear that the FSS can be ‘locked' strictly below 1 μeV, that is, high-fidelity entangled photons can be generated from the QDs, for a large range of exciton emissions. Considering the typical lifetime (500 ps–1 ns) of our QDs and therefore a radiative limited linewidth of ∼1 μeV, the tuning range of 3.7 meV (2.3 nm) shown in Fig. 3a corresponds to >3,000 times of the radiative linewidth. This tunability is >1 order of magnitude larger than what has been demonstrated for SPDC sources^32^.

We have performed the polarization cross-correlation spectroscopy^11^,^14^,^15^,^17^ on a brighter QD-embedded inside another device on the same chip. The FSS is tuned to around zero (0.21±0.20 μeV) to demonstrate the polarization entanglement, and the data are presented in Fig. 3b. A key criterion for entanglement is the presence of a correlation independent of the chosen polarization basis, that is, 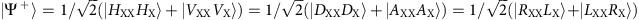, with D, A, R, L denoting the diagonal, anti-diagonal, right-hand circular and left-hand circular polarizations. Clear photon bunching, with a normalized second-order correlation function g^(2)^(*τ*=0)>3, can be observed for the co-polarized HH and DD photons, whereas in the circular basis the bunching occurs for the cross-polarized RL photons. The entanglement fidelity *f*^*+*^ to the maximally entangled Bell state can be determined from the measurements in Fig. 3b, see Methods section. The peak near the zero time delay yields a fidelity *f*^*+*^ of 0.733±0.075 without any background subtraction, which exceeds the threshold of 0.5 for a classically correlated state by >3 s.d. The above results are in line with previous experimental and theoretical works, and verify that highly entangled photons can be generated with our device with large wavelength tunability.

**Figure 4:**
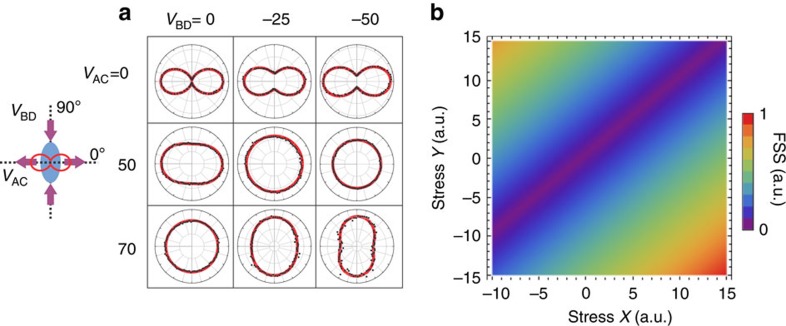
Semi-quantitative description of the independent tunability of exciton wavelength and FSS. (**a**) Matrix of polarization plots at different voltage combinations. Here the exciton energy is plotted as a function of *θ*, with the same definition being used in the inset of Fig. 2a. All polar plots have an axis scale of 15 μeV, and the solid red lines represent a fit to the data with a sinusoidal function. The ‘circularity' and the direction of the pedals indicate the relative amplitude of FSS and *θ*, respectively. Circular polarized exciton emission with zero FSS can be observed at three different voltage combinations, that is, at different exciton emission wavelength. No appreciable polarization rotation can be observed at the applied voltages. The schematic shows the stress condition for this type of QDs. (**b**) A density plot of the FSS as a function of the two stress magnitudes *X*,*Y* as obtained with the two-level model Hamiltonian. FSS is kept at zero for a range of stress combinations (*X*,*Y*), therefore the wavelength at which FSS is zero can be tuned at will.

An intuitive understanding of the tuning behaviour can be obtained immediately from a matrix of exciton polarization plots at different voltage combinations, Fig. 4a. The ‘circularity' of the polarization pedals indicates the relative magnitude of FSS. By sweeping *V*_AC_ from 0 to 70 V at *V*_*BD*_ of 0 V, an increasing tensile stress is applied along legs A&C and the symmetry is gradually recovered. We observe the ‘opening' of the polarization pedal without any appreciable rotation^19^,^20^,^31^, as predicted by theory^29^. At the voltage combination (*V*_AC_, *V*_BD_)=(70, 0) *V*, the exciton emissions become circular polarized with a near-zero FSS and polarization-entangled photons are generated. At *V*_BD_ of −25 V and −50 V, the symmetry is already partially recovered due to the increased compressive stress along the legs B&D, and therefore the symmetry can be fully recovered with less tensile stress, that is, a smaller *V*_AC_, along the legs A&C. Due to the symmetry of orthogonal uniaxial stresses, we can observe the same effect when sweeping *V*_AC_ at fixed voltages of *V*_BD_.

Our experimental findings can also be semi-quantitatively understood using the recent theoretical observation, Supplementary Note 1 (ref. 26). The application of two orthogonal stresses of magnitude *X* and *Y* can be used as effective knobs to erase the FSS and tune the exciton emission once the stress axis are aligned with the initial exciton polarization. In this case indeed, the FSS scales linearly with the stress anisotropy Δ=*X*–*Y* and eventually vanishes at a critical stress anisotropy Δ=Δ*c*. This behaviour is demonstrated in Fig. 4b where we show a density plot of the FSS as a function of the two stress magnitudes *X*,*Y* as obtained with the two-level model Hamiltonian, Supplementary Note 1 (refs 26, 29). Furthermore, the remaining independent hydrostatic part ∝ *X*+*Y* can be used to change at will the exciton wavelength *λ*_X_ (energy *E*_X_). This implies a linear interdependence between the exciton emission and the FSS, which is in perfect agreement with the data presented in Fig. 2.

We emphasize that the device presented here holds strong promise for the realization of solid-state scalable QIP platform. First, the wavelength tunability allows the Bell state measurement between two QDs and therefore the swapping of entanglement^24^,^33^. Also with suitable QDs (see for example Fig. 1d), it is straightforward to couple these sources with atomic vapours^34^. Second, the integrated MEMS on silicon have small footprints and low operation voltages, which solves two of the most challenging problems of the strain engineering technique^15^,^25^. We can foresee the integration of this device with other photonic structures (for example, with circular ring grating cavities to enhance the photon collection efficiency^35^) and, most intriguingly, with advanced silicon quantum photonic circuits. The envisioned hybrid can exploit the maturity of CMOS technology, and as well as the waveguiding, processing and detection capabilities associated with silicon photonics.

In summary, we have experimentally realized wavelength-tunable entangled photon sources on a III–V/Si chip, which represents an important step towards scalable entangled photon sources based on III–V QDs. The reported device will play an important role not only in building a solid-state quantum network based on entanglement swapping and quantum memories, but also in building advanced quantum photonic circuits for on-chip QIP applications. The MEMS based device features the advantages of sophisticated anisotropic stress control on chip. We envision that it will inspire many other topics in quantum and nano-technologies, and an interesting perspective is to replace the nanomembranes with the emerging two-dimensional materials and to study the strain-dependent photonic and electronic properties.

## Methods

### Sample growth

The studied sample was grown on a (001) GaAs substrate by solid-source molecular beam epitaxy. Several samples were used in this work, but their general structures are the same. After the deoxidization and GaAs buffer growth, a thin layer of Al_0.75_Ga_0.25_As was grown as the sacrificial layer for the nanomembrane release. The InAs QDs were grown by partial capping and annealing, and embedded in the middle of a GaAs layer with a thickness of a few hundred nanometres. The emission range of the QDs is between 880 and 900 nm and we can easily identify low-density regions with <1 QD μm^−2^.

### Device fabrication

As for the device processing, we start with the QD-embedded nanomembranes. Standard UV photolithography and wet-chemical etching were used to fabricate mesa structures with a size of 80 × 80 μm^2^. The edge of the nanomembranes was processed along [110] or [1–10] crystal axis of GaAs. The PMN-PT film is ∼15 μ thick. The backside is coated with gold as the bottom/ground contact. And the PMN-PT film is bonded to a silicon substrate via glue (Ibule photonics). We use focused ion beam to define the trenches in the film, and the depth of the trenches is deep enough to penetrate into the silicon substrate. And then the top contact is deposited by E-beam sputtering. After this, the device is undercut by wetting chemical etching to form the suspended PMN-PT thin-film legs.

Then we bond the QD-embedded nanomembranes to the processed PMN-PT/Si substrate using a flip-chip bonder. The edge of nanomembrane is carefully aligned along the strain axis of the PMN-PT actuation legs. To constrain the displacements of the membrane, the same voltage is applied to opposite legs.

### Optical measurement

For optical measurements, the device is loaded into a cryostat chamber which is cooled to ∼5 K. The PL emitted from the QDs is collected by a × 100 microscope objective with numerical aperture of 0.52. The signal is then dispersed by a spectrometer with 1,800 grating and detected by a liquid nitrogen cooled CCD. By inserting a half-wave plate and a linear polarizer directly after the collection lens, polarization-resolved measurements were performed to estimate the FSS. The exciton polarization is determined by aligning the fast optical axis of the polarizer along [1–10] direction of the nanomembrane. With the experimental procedure used in previous works^13^,^26^, we can determine the FSS with an accuracy of sub-μeV. For FSS of below 1 μeV, an error bar of ±0.25 μeV is extracted from the 95% prediction band of the sine fit function. As for the polarization correlation spectroscopy, a non-polarizing 50:50 beam splitter is placed directly after the objective to divide the optical paths between two spectrometers, which are then used to detect X and XX separately. After each spectrometer, a Hanbury Brown-Twiss set-up, consisting of a polarizing beam splitter and two high-efficiency single-photon avalanche detectors, is placed. Half- and quarter-wave were used to select the proper polarization basis. The temporal resolution of the system is ∼450 ps. The entanglement can be quantified by measuring degree of correlation *C*, which s defined by


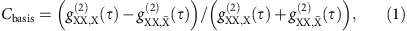


where 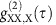 and 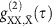 are normalized second-order time correlations for co-polarized and cross-polarized XX and X photons, respectively. The fidelity *f*^+^ is calculated by using the formula: *f*^+^ =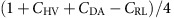, in which *C*_HV_, *C*_DA_ and *C*_RL_ are degree of correlations in HV, DA and RL bases.

## 

## Additional information

**How to cite this article:** Chen, Y. *et al*. Wavelength-tunable entangled photons from silicon-integrated III–V quantum dots. *Nat. Commun.* 7:10387 doi: 10.1038/ncomms10387 (2016).

## Supplementary Material

Supplementary Information Supplementary Figures 1-2, Supplementary Note 1 and Supplementary References.
