# Supplementary material for: Wavelength-tunable entangled photons from silicon-integrated III–V quantum dots
Source: Nat Commun. 2016 Jan 27;7:10387. doi: 10.1038/ncomms10387 (PMC4737807; doi:10.1038/ncomms10387)
Supplement: Supplementary Information — Supplementary Figures 1-2, Supplementary Note 1 and Supplementary References. [file ncomms10387-s1.pdf]

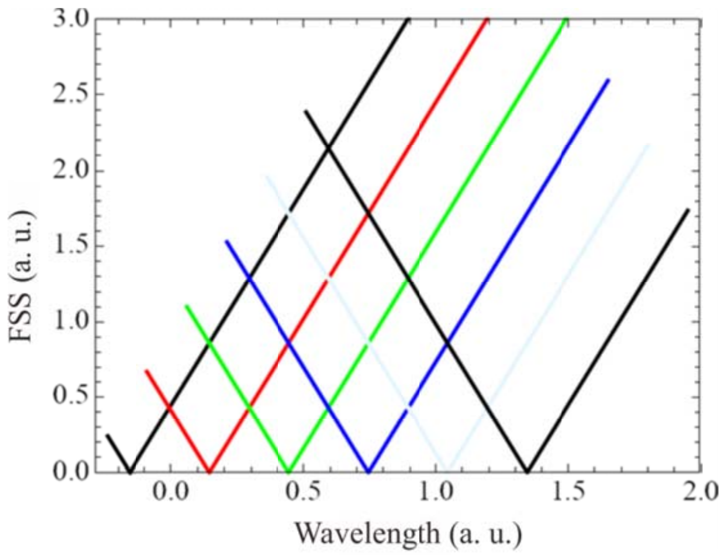

Supplementary Figure 1: Theoretical modelling of the independent tunability of exciton wavelength and FSS with orthogonal stress.

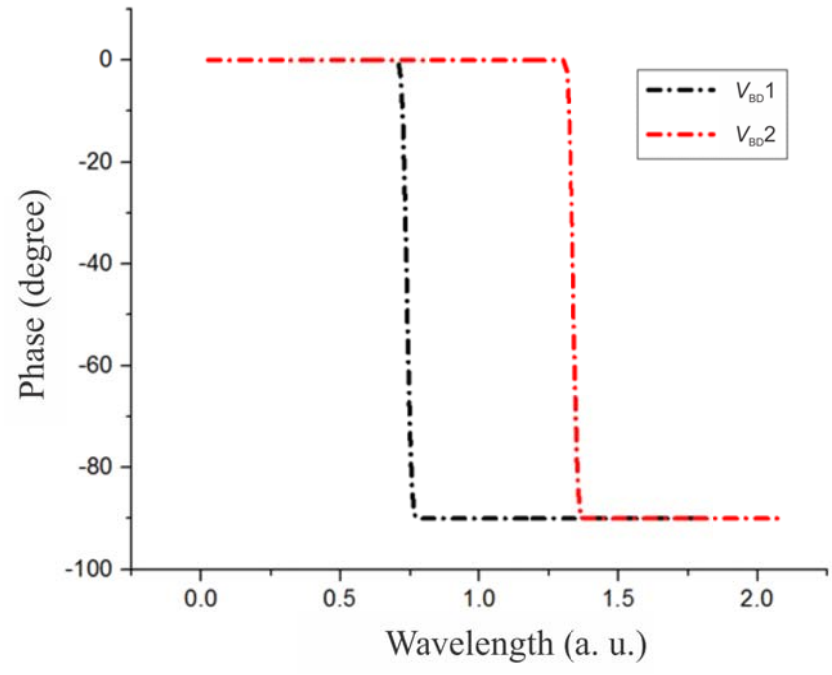

Supplementary Figure 2: Theoretical modelling of the phase rotation of exciton when the FSS is tuned across zero point. One pair of leg *e.g.* V_{BD} is biased at different voltages. The phase rotation is always 90 degree for both cases.

Supplementary Note 1: Theory of anisotropic strain-engineering of semiconductor quantum dots

The starting point of this theory consideration based on k · p theory,[1] is the presence of a four-leg piezoelectric that preduces two orthogonal uniaxial stresses, written as $\sigma_{A,B}$. We assume the legs of the actuator to be at a given angle α with respect to the [110] and [1-10] directions. The first thing to do is to express the stresses along the [100] and [010] crystallographic directions. This can be accomplished by making a simple rotation of an angle $\pi/4+\alpha$. The resulting biaxial stress then has the following form

$$\sigma_{xx} = \frac{\sigma_A+\sigma_B}{2} - \frac{\sigma_A-\sigma_B}{2}\sin 2\alpha \quad (1)$$

$$\sigma_{yy} = \frac{\sigma_A+\sigma_B}{2} + \frac{\sigma_A-\sigma_B}{2}\sin 2\alpha \quad (2)$$

$$\sigma_{xy} = \frac{\sigma_A-\sigma_B}{2}\cos 2\alpha \quad (3)$$

Using the stress-strain relation for a conventional cubic material, we have for the strain tensor

$$\epsilon_{xx} = (S_{11}+S_{22})\frac{\sigma_A+\sigma_B}{2} - (S_{11}-S_{22})\frac{\sigma_A-\sigma_B}{2}\sin 2\alpha \quad (4)$$

$$\epsilon_{yy} = (S_{11}+S_{22})\frac{\sigma_A+\sigma_B}{2} + (S_{11}-S_{22})\frac{\sigma_A-\sigma_B}{2}\sin 2\alpha \quad (5)$$

$$\epsilon_{xy} = S_{44}\frac{\sigma_A-\sigma_B}{2}\cos 2\alpha \quad (6)$$

$$\epsilon_{zz} = S_{12}(\sigma_A+\sigma_B) \quad (7)$$

In order to evaluate strain effects on the exciton Hamiltonian of the quantum dot, we take into account only the anisotropic strain effects on the holes. One starts with the Dresselhaus-Kip-Kittel Hamiltonian,[1] for$|X\rangle$, $|Y\rangle$, $|Z\rangle$ p-orbitals and applies the Bir-Pikus formalism. With this the strain part of the Hamiltonian is

$$H_{DKK}^{BP} = \begin{pmatrix} l\epsilon_{xx}+m(\epsilon_{yy}+\epsilon_{zz}) & n\epsilon_{xy} & 0 \\ n\epsilon_{xy} & l\epsilon_{yy}+m(\epsilon_{yy}+\epsilon_{xx}) & 0 \\ 0 & 0 & l\epsilon_{zz}+m(\epsilon_{yy}+\epsilon_{xx}) \end{pmatrix} \quad (8)$$

Where l, m, n are the deformation potentials and the strain components are those written above. This is not complete yet since the strain Hamiltonian for the heavy holes are also needed. It can

be obtained from the Hamiltonian above making a $\pi/4$ counterclockwise rotation. The corresponding block for the heavy holes then can be simply expressed in terms of Pauli matrices τ as[2]

$$H_{\text{strain}} = \bar{\beta}(\sigma_A + \sigma_B)\tau_0 + \beta(\sigma_A - \sigma_B)\cos 2\alpha\tau_z + \gamma(\sigma_A - \sigma_B)\sin 2\alpha\tau_x \quad (9)$$

where $\bar{\beta}$, β and γ are parameters related to the elastic constants renormalised by the deformation potentials. Next we have to take into account the exciton Hamiltonian. The latter can be again written in terms of Pauli matrices as

$$H_{e-h} = E_0\tau_0 + \eta\tau_z + \kappa\tau_x \quad (10)$$

Where η accounts for the FSS for C_{2v} quantum dots while the parameter κ accounts for the further reduction to C_1 due to alloying effects.[3] The full Hamiltonian is of course then given

$$\mathrm{H} = H_{e-h} + H_{\text{strain}} \quad (11)$$

Following the work by Gong *et al.*,[4] diagonalization of the above Hamiltonian yields the eigenvalues and the eigenvectors. We also write down the FSS for orthogonal uniaxial stresses with $\sigma = \sigma_A - \sigma_B$,

$$\mathrm{FSS} = 2\sqrt{(\beta\sigma\cos(2\alpha) + \eta)^2 + (\gamma\sigma\sin(2\alpha) + \kappa)^2} \quad (12)$$

For the aligned dots, the following relation holds:

$$\tan(2\alpha) = (\beta/\gamma) * (\kappa/\eta) \quad (13)$$

and, the FSS formula is reduced to a simple case.

$$\mathrm{FSS} = \frac{2(\beta\sigma\cos(2\alpha) + \eta)}{\eta} * \sqrt{\eta^2 + \kappa^2} \quad (14)$$

This implies that multiple zero points of FSS can be achieved by adjusting the difference between the orthogonal uniaxial stresses. Therefore the independent tuning of the two orthogonal uniaxial stresses yields a line of zero FSS. To demonstrate this, we make a general plot for the FSS and polarization angle against the wavelength which is shown in Supplementary Figure 1.

The tuning behavior of FSS is within remarkable agreement with our experimental finding and indicates the ability of control the FSS and the wavelength independently. And also we plot the change of exciton polarization when FSS is tuned across zero. From Supplementary Figure 2, we see that the polarization of the exciton is always rotated by 90 degrees when legs B&D is biased

at different voltages. And the 90 degree phase rotation, on the other hand confirms our ability to eliminate the FSS.

Supplementary Reference

1. Voon LCLY, Willatzen M. *The k p Method*. Springer-Verlag Berlin Heidelberg (2009).

2. Trotta R, Martín-Sánchez J, Daruka I, Ortix C, Rastelli A. Energy-Tunable Sources of Entangled Photons: A Viable Concept for Solid-State-Based Quantum Relays. *Physical Review Letters* **114**, 150502 (2015).

3. Mlinar V, Zunger A. Effect of atomic-scale randomness on the optical polarization of semiconductor quantum dots. *Physical Review B* **79**, 115416 (2009).

4. Gong M, Zhang W, Guo G-C, He L. Exciton Polarization, Fine-Structure Splitting, and the Asymmetry of Quantum Dots under Uniaxial Stress. *Physical Review Letters* **106**, 227401 (2011).
